# Cost-effectiveness of malaria microscopy and rapid diagnostic tests *versus *presumptive diagnosis: implications for malaria control in Uganda

**DOI:** 10.1186/1475-2875-10-372

**Published:** 2011-12-19

**Authors:** Vincent Batwala, Pascal Magnussen, Kristian S Hansen, Fred Nuwaha

**Affiliations:** 1 Department of Community Health, Mbarara University of Science & Technology P. O. Box 1410, Mbarara, Uganda; 2 Centre for Health Research and Development, Faculty of Life Sciences, Copenhagen University, Thorvaldsensvej 57, DK1871 Frederiksberg C, Denmark; 3 Department of Global Health and Development, London School of Hygiene and Tropical Medicine, 15-17 Tavistock Place, London WC1H 9SH, UK; 4 Disease Control and Environmental Health, Makerere University School of Public Health, P. O. Box 7072, Kampala, Uganda

## Abstract

**Background:**

Current Uganda National Malaria treatment guidelines recommend parasitological confirmation either by microscopy or rapid diagnostic test (RDT) before treatment with artemether-lumefantrine (AL). However, the cost-effectiveness of these strategies has not been assessed at rural operational primary care centres.

**Methods:**

Three health centres (HCs) were randomized to three diagnostic arms (microscopy, RDT and presumptive diagnosis) in a district of low and another of high malaria transmission intensities in Uganda. Some 22,052 patients presenting with fever at outpatients departments were enrolled from March 2010 to February 2011. Of these, a random sample of 1,627 was selected to measure additional socio-economic characteristics. Costing was performed following the standard step-down cost allocation and the ingredients approach. Effectiveness was measured as the number and proportion of patients correctly diagnosed and treated. Incremental Cost-Effectiveness Ratios (ICERs) were estimated from the societal perspective (http://Clinicaltrials.gov, NCT00565071).

**Results:**

Overall RDT was most cost-effective with lowest ICER US$5.0 compared to microscopy US$9.61 per case correctly diagnosed and treated. In the high transmission setting, ICER was US$4.38 for RDT and US$12.98 for microscopy. The corresponding ICERs in the low transmission setting were US$5.85 and US$7.63 respectively. The difference in ICERs between RDT and microscopy was greater in the high transmission area (US$8.9) than in low transmission setting (US$1.78). At a willingness to pay of US$2.8, RDT remained cost effective up to a threshold value of the cost of treatment of US$4.7.

**Conclusion:**

RDT was cost effective in both low and high transmission settings. With a global campaign to reduce the costs of AL and RDT, the Malaria Control Programme and stakeholders need a strategy for malaria diagnosis because as the cost of AL decreases, presumptive treatment is likely to become more attractive.

## Background

The replacement of conventional anti-malarial drugs with artemisinin-based combination therapy (ACT) for treatment of uncomplicated malaria stimulated the interest in reassessing the diagnostic practices in malaria-endemic countries in sub-Saharan Africa [1-3]. Clinical (presumptive) diagnosis of malaria, the current diagnostic strategy in remote settings leads to considerable drug expenditure [4,5] on inappropriate treatment of non-parasitaemic patients [6-8]. The World Health Organization and the Uganda national guidelines [9,10] recommend parasitological confirmation of malaria either by microscopy or rapid diagnostic tests (RDT) before treatment is started.

In rural health centres (HCs) routine malaria microscopy if available, is often of limited quality [11,12]. In view of the limitations of microscopy, Uganda commenced the rollout of malaria RDTs (primarily histidine-rich protein II [HRP2] based tests) in parish and sub-county level HCs with no laboratory infrastructure, as a means of targeting ACT use. The rollout was phased commencing with six districts in 2008. The exercise is however stalled due to stock-out of RDTs from end of 2009. With availability of RDTs, scaling up is planned to an additional 22 districts. Currently, there are 112 districts in the country.

There is considerable debate regarding the cost-effectiveness of routine use of RDTs as an integral part of deploying ACTs. On the one hand, some studies conducted elsewhere reported that replacement of malaria microscopy with RDTs would increase the provider costs [2,13]. On the other hand, relative to microscopy, the use of RDTs was likely to be cost-effective [3,14-16]. In the face of the dynamic market, this debate necessitated an empirical economic evaluation of the available malaria diagnostic techniques in the local setting. The current study assessed the cost effectiveness of treating malaria with artemether-lumefantrine (AL) based on microscopy, RDT or presumptive diagnosis at sub-county public HCs from the societal perspective.

## Methods

### Study setting

The study was carried out in six sub-county level government HCs, three in Bushenyi and three in Iganga districts of Uganda. Bushenyi district is located in south-western Uganda, 310 km from the capital city Kampala. HCs in Bushenyi are located at an altitude of 1,744-1,962 m above sea level. The climate is relatively wet with annual rainfall of 800-2,000 mm, and annual temperature range of 12.5°-30°C. The substantial part of Bushenyi is of hilly-rough and rugged terrain with the topography dominated by undulating hills. Iganga district is located in eastern Uganda. The land surface is mainly flat. HCs in Iganga are located at an altitude of 1,059-1,119 m above sea level. The temperatures range from 23°-27°C with annual rainfall of 900-1,200 mm. The Uganda government categorizes Bushenyi as low and Iganga as high malaria transmission intensity settings. Since 2001, patients do not pay for medical services at public HCs. Bushenyi and Iganga were not among the districts in the first phase of RDT rollout. However, RDTs were introduced in the "RDT arm" by this trial in 2007. Additional description of the study setting was published elsewhere [17].

### Study design and population

The study was a randomized cost-effectiveness trial (http://Clinicaltrials.gov: NCT00565071). In each district, HCs were randomized to three diagnostic arms (microscopy, RDT and presumptive diagnosis). The study population included HC clinical and laboratory staff and all outpatients presenting with fever (by statement or measured axillary temperature ≥37.5°C). Of these, a random sample of 1,627 outpatients was chosen to measure selected socio-economic data. The study was integrated within the existing district health services delivery system to reflect the real-life operational conditions.

### Laboratory procedures

The detailed description of the laboratory procedures and subsequently the performance (sensitivity and specificity) of the three diagnostic strategies has been published [12]. Briefly, finger-prick blood specimens were drawn from a sample of 300 outpatients attending HCs stated above. They were then processed following standard operating procedures. The validity indicators were gauged against polymerase chain reaction as reference standard.

### Collection of cost data

Cost data was categorized into internal costs (public provider costs) and external costs (patient costs). Data were collected from March 2010 to February 2011 as detailed below.

### Collection of internal costs

Real-life facility cost data were collected covering a period of 12 months in which 22,052 malaria visits were made. The data included recurrent costs (personnel, stationery, utilities, medicines, clinical and laboratory supplies) and capital costs (buildings, equipment and furniture). Financial reports were reviewed and interviews were conducted with staff to ascertain additional resources used such as the primary health care funds and those likely to be donations. Costs were collected in Uganda shillings (Ush) and converted to US dollars (exchange rate US$1 = Ush2,380, February 3^rd ^2011).

### Health centre personnel costs

The effective contact time with those seeking care was one of the main input parameters for personnel costs. Contact time was recorded for a random sample of 1,627 outpatients. When a patient arrived at the HC, time was recorded by the research assistant on a "time sheet." The "time sheet" was then given to the patient. Thereafter, the time was recorded by clinicians and laboratory personnel for every service provided. The "time sheet" was finally retained at the dispensing window. For the laboratory personnel, the effective contact time was comprised of: drawing a sample from the patient, slide preparation, scanning the 200 film fields until declaring a slide negative and reporting of results. With regard to RDT, effective contact time was comprised of: drawing blood samples from patients, applying samples onto the test, test reading and reporting of results. The outpatient clinics at sub-county HCs run for eight hours from Monday to Saturday and about five hours on Sunday. Therefore, HC staff work 68 hours (4,080 minutes) weekly. Assuming a year of 52 weeks; HC staff work 48 weeks, less four weeks of annual leave. The HC staff were also interviewed regarding their time allocation for other services. The time used for administration was documented separately by the research assistants based at the HCs. Personnel monthly salaries were recorded from their pay-slips.

### Drugs, laboratory supplies and RDT

Artemether-lumefantrine was costed as it is distributed in four different fixed-dose weight-specific packs (35 kg and above, 25-34.9 kg, 15-24.9 kg and 5-14.9 kg). The costing of non-malaria treatment was based on "per tablet" or "per capsule" of prescribed drugs to make up a dose in each of the weight categories. All laboratory supplies used for the 12 months were documented. The cost of RDT (Paracheck, Orchid Biomedical Systems, Goa, India) was US$0.84 as per Joint Medical Stores price catalogue. Medicines and supplies used were recorded from primary source documents including dispensary records, outpatient registers, laboratory records and stock cards. The cost of medicines and supplies were obtained from Uganda National Medical Stores and Joint Medical Stores delivery reports kept at the HCs. Medicines and disposables that were supplied but not used during the study period were excluded.

### Utilities and stationery

Utilities mainly included coffee/tea for staff, water and fuel for lighting. None of the six HCs had electricity at the time of implementation of this study. The water source was either bore-hole or rain-harvested. The quantity of water used per week in litres was annualized and converted into National Water and Sewerage Corporation (NWSC) units. Costing of water was performed basing on NWSC institutional/government rate [18]. The costs of fuel, sugar and coffee/tea were obtained from receipts and HC reports. The health management information system registers, laboratory registers and stock cards were mainly supplied by National Medical Stores and the costs were obtained from delivery reports. The costs of additional stationery purchased were estimated from market prices.

### Capital costs

Capital goods were those items with a useful lifespan of more than one year (buildings, equipment and furniture). Inventories of capital goods were generated and grouped within their locations (rooms) in the HC. HC building plans were obtained from the district engineers' office to determine the construction costs and relative contribution of area coverage to outpatient case management. The costs of renovations and repairs (if any) within the 12 months of the study were also collected.

### Collection of patient (external) costs

Patient costs were episode-related expenditures. A sample of 1,627 outpatients was enrolled for collection of external costs. On arrival at the HC, the time was recorded on a "time sheet" but patients were also asked to state the estimated time of departure from home. Patients were systematically tracked until departure from the HC. The aim of tracking patients was to estimate the time spent accessing services. Exit interviews were carried out and information recorded on study questionnaire. The patient travel time was equal to arrival time minus the estimated time of departure from home. It was assumed that patients used a similar time for the return journey. The duration of interviews (in minutes) was eliminated during analysis. Patient costs were categorized into direct (out-of-pocket expenses on transport and other incidentals related to treatment seeking) and indirect (lost income due to medical care seeking, including travel and waiting time). Direct patient costs were valued according to reported expenditures. Because majority of the patients were rural and mainly peasants, lost earnings due to care-seeking were valued using the Uganda Ministry of Public Service minimum wage "for unskilled labour" salary scale U8 "entry point for support staff mainly attendants" which is approximately US$55.9 per month employing standard methods [19].

### Valuation of resource use and unit cost of diagnosis for each outpatient visit

The unit cost of diagnosis for each outpatient visit in each arm was determined following a standard step-down cost allocation method [20,21] of all available HC resources. The ingredients technique was also employed and provided data directly measured for example "service provider effective contact time" with patients, quantity and costs of drugs and supplies used. Further, the annualized values of buildings, furniture and equipment were estimated using a standard procedure [21] assuming a useful lifespan for these goods of 30, 10 and seven years respectively and with a discount rate of 3%. Three major cost centres (overhead, support and the final services) were identified. Resources (Table 1) were allocated in three steps separately for each HC and later aggregated. In the first step, the total cost of running each HC were allocated to the three cost centres. Personnel salaries were allocated using the measured staff time. Laboratory supplies, cleaning materials, drugs and disposables were proportioned or fully allocated to the relevant cost centres. Capital costs and stationery were allocated basing on actual or estimated use. In the second step, overhead costs were allocated to the support and final services centre. In the third step, the total costs of running a HC were subsequently allocated to the level performing the individual final services, such as the outpatient department (OPD) using an allocation criterion that reflected the actual resources used. The total costs of running an OPD were divided by the number of final services to arrive at the unit cost per service with and without parasitological confirmation of malaria.

**Table 1:** Aggregate resources (US$) at two health centres per arm and allocation criteria

Recurrent costs	Presumptive	RDT	Microscopy	Allocation criteria
Salaries	24955	28374	40001**^§^**	Measured staff time
Drugs and disposables	13757	14177	18521	To relevant cost centre
Stationery	311	393	432	Estimated use
Utilities	459	393	565	Relative to size of space
Laboratory and RDT supplies	N/A	4221	1874	To relevant cost centre
Cleaning materials	340	306	331	To relevant cost centre
Capital costs*				
Building**	3749	5973	7497	Actual use
Equipment	402	498	767	Actual use
Furniture	144	149	216	Actual use

Total	44,117	54,484	70,204	

N/A = not applicable, RDT = rapid diagnostic test

**^§^**includes salaries of laboratory personnel

*goods with useful lifespan of more than one year, costs annualised at 3% discount rate

** includes sits in the waiting area made of fixed concrete

### The cost effectiveness analysis (CEA) model

In order to determine the cost effectiveness, a decision tree in TreeAge was used with a patient cohort presenting at the outpatient department of a rural HC. Patients entered the model at the time when the attending clinicians suspected uncomplicated malaria. Once in the model, patients were investigated using microscopy, RDT or presumptive diagnosis. Effectiveness values of the strategies (Table 2) and costs of treating an outpatient (provider and patient costs combined) were used to populate the model. Because there is normally over prescription of analgesics, it was assumed that a true parasitaemic patient getting AL was also likely to get an analgesic. It was also assumed that a patient with negative test result got antibiotic and analgesic treatments.

**Table 2:** Comparison of effectiveness of the three diagnostic strategies

Test results^ψ^	Diagnostic strategy		
	
	Presumptive	Microscopy	RDT
True positive^a^	35	42	81
False positive	53	14	29
False negative	54	47	8
True negative^b^	158	197	182
Total^c^	300	300	300
Number correctly diagnosed^d^	193	239	263
Proportion correctly diagnosed (%)^e^	64.3	79.7	87.7

^ψ^gauged against polymerase chain reaction as reference standard, RDT = rapid diagnostic test, d = (a+b), e = (d/c)*100

### Statistical analysis

Data was double-entered and validated in EpiData version 3.1 software (The EpiData Association, Odense, Denmark) and analysed in Stata version 10 (Stata Corp, College Station, Tx, USA), TreeAge and MS-Excel. STATA was mainly used in the descriptive analysis. Excel was used in costing especially Step-down accounting. TreeAge was used for constructing and analysing the decision tree and uncertainty in the cost-effectiveness model. The *p*-values were calculated at the 0.05 level of significance and Confidence Interval (CI) was set at 95%.

### Sensitivity analysis

Sensitivity analysis was conducted in TreeAge on the variables that were uncertain and prone to change over time. These included prevalence of malaria, costs of RDT and AL. Sensitivity and specificity [12] were also examined. The cost of AL and RDT were halved and doubled to get the lower and upper limits of the interval respectively.

### Outcome measures

The primary endpoints were: 1) effectiveness measured as the number and proportion of patients correctly diagnosed (true positive + true negative) and treated; and 2) incremental cost per additional case correctly diagnosed and treated (incremental cost effectiveness ratio - ICER) defined as the change in costs over the change in effectiveness of moving from the presumptive strategy (the base case) to the next best alternative.

### Ethical approval

The study was approved by Makerere University School of Public Health Higher Degrees Research and Ethics Committee; and the Uganda National Council for Science and Technology (Ref: HS 209). The study was registered with the http://Clinicaltrials.gov (NCT00565071).

## Results

### Effectiveness of the three diagnostic strategies

Effectiveness of each diagnostic strategy was calculated based on a recent publication [12] from the same trial preceding this paper. Malaria RDT was the most effective with the number of cases correctly diagnosed and treated being 263, followed by microscopy: 239 and presumptive diagnosis: 193 (Table 2). The corresponding proportions of patients correctly diagnosed and treated were 87.7%, 79.7% and 64.3% respectively. Routine use of RDT or microscopy would result into an additional 36.3% and 23.8% respectively of patients correctly diagnosed and treated in comparison to the presumptive technique.

In the high transmission area, effectiveness values were: RDT 86.0%, microscopy 70.7% and presumptive diagnosis 59.3%. The corresponding effectiveness values in the low transmission setting were 89.3%, 88.7% and 69.3% respectively.

### Analysis of internal costs

The unit cost of diagnosis in United States dollars (US$) in each arm was determined using the number of final services in the outpatient department. Overall, 22,052 malaria visits were made over the 12 months of data collection period. The costs for each strategy were allocated using the step-down technique to the level of an individual attending the OPD. The cost analysis for each strategy is presented in Table 3 and briefly described as follows:

**Table 3:** Aggregate and unit cost of diagnosis by diagnostic strategy (US$)

	Presumptive	Microscopy	RDT
Total cost allocation for OPD services	18807	31759	25437
Number of final services at OPD	20103	24864	18480
Total cost allocation for management of malaria at OPD	10242	16553	8512
Number of OPD malaria visits made in the 12 months^a^	10446	7560	4046
Total cost allocation for OPD malaria diagnostic services^b^	6480	9515	5227
Number of OPD malaria tests done^c^	N/A	6219	4039
Unit cost per malaria test^d^	N/A	1.53	1.29
Unit cost of presumptive diagnosis^e^	0.62	N/A	N/A

RDT = rapid diagnostic test, OPD = outpatient department, N/A = not applicable, d = (b/c), e = (b/a)

#### The presumptive strategy

The overall cost allocation for the outpatient services in the presumptive arm was US$18,807 (Table 3). There was no parasitological confirmation of malaria in this arm. Therefore, the total allocation for clinical diagnosis of malaria was equal to the total for malaria management in the OPD (US$10,242) minus that of drugs and disposables (US$3,762) which was equal to US$6,480. The aggregate number of malaria visits in the two HCs constituting the presumptive arm was 10,446. Therefore, the unit cost of presumptive diagnosis was US$6,480/10,446 which is equal to US$0.62.

#### Rapid diagnostic test strategy (RDT)

In the RDT arm, the total cost of running an OPD was US$25,437 (Table 3). Out of this, US$12,100 (47.6%) was allocated to management of malaria, of which US$5,227 was for diagnostic services. The cost of RDTs was the major determinant constituting 74.6% of the amount allocated to diagnostic services. The aggregate number of malaria RDTs performed in the two HCs in this arm was 4,039. Therefore, the unit cost of diagnosis was US$1.29, which was lower than that for HC microscopy.

#### The microscopy strategy

The total cost of running an OPD was US$31,759, with US$16,553 (52.1%) allocated to malaria management of which US$9,515 (57.5%) was to diagnostic services. Laboratory supplies and equipment accounted for 17.9% while salary constituted 56.7% of the diagnostic services. The number of malaria investigations done over the period of study was 6,219 giving a unit cost of diagnosis of US$1.53.

### Drug costs

The unit cost of AL fixed-dose weight-specific pack decreased with the weight of the patient. However, the average cost of AL dose was US$1.38. The average cost of antibiotic/analgesic dose was US$1.05 and the pattern across the weight-specific groups was similar to that of AL.

### External costs (patient costs)

In public HCs, patients do not pay for medical services. Therefore, patient costs relate to direct out-of-pocket expense on the episode prior to visiting the HC, transport and other non-medical incidentals (external direct costs); and the indirect cost of travel and waiting time (external indirect costs).

### External indirect costs

The mean distance from home to the HC was 5.7 km [5%CI: 2.9-8.4]. Overall, HC staff in the presumptive arm tended to report to work in late morning after patients had arrived resulting into an extended visiting time. The mean travel time was 139.5 minutes [95%CI: 131.1-147.9], but was not different between arms (Table 4). The time spent accessing services when using RDT 134.4 minutes [95%CI: 123.5-145.3] was not different from that of presumptive treatment, but significantly shorter than that of microscopy 188.5 minutes [95%CI: 173.8-203.3]. The travel time and the overall time for the HC visit were converted into a single monetary measure "opportunity cost." This implies that on average, the time cost due to absence from work as a result of suffering from suspected or confirmed malaria was US$1.59. The time cost with subsequent loss of income was likely to be greater in the microscopy arm (US$1.68) compared to RDT US$1.45, *p *< 0.001, or presumptive-based treatment US$0.80, *p *= 0.005 because the time taken to diagnose a case and produce results was much longer in the microscopy arm.

**Table 4:** Mean direct expenditure and lost income while seeking care (US$) at government health centres

Variable	Overall Mean[95%CI]	Presumptive Mean[95%CI]	RDT Mean[95%CI]	Microscopy Mean[95%CI]
Monthly household income	19.8[15.0-24.5]	21.4[11-31.8]	16.5[9.4-23.6]	21.3[14.9-27.7]
Medication prior to visiting HC	0.40[0.34-0.46]	0.44[0.32-0.56]	0.40[0.27-0.52]	0.34[0.30-0.44]
Expense on transport	0.43[0.35-0.50]	0.43[0.18-0.69]	0.39[0.26-0.52]	0.44[0.34-0.54]
Out-of-pocket (other non-medical) expense	0.20[0.11-0.29]	0.30[0.06-0.66]	0.13[0.03-0.22]	0.23[0.12-0.34]
Opportunity cost (travel and waiting)	1.59[1.53-1.66]	0.80[0.71-0.94]*	1.45[1.35-1.50]	1.68[1.57-1.79]

*only travel time converted into money, CI = Confidence Interval, HC = health centre, RDT = rapid diagnostic test

### External direct costs

The mean monthly household income was US$19.8 [95%CI: 15.0-24.5] (Table 4), and was similar in the three arms. The mean expenditure on treating the episode prior to visiting the study HC was US$0.40 [95%CI: 0.34-0.46], still not significantly different between the study arms. Patients in the low transmission setting significantly incurred direct expense on transport US$0.65 [95%CI: 0.55-0.76], compared to those in the high transmission area US$0.31 [95%CI: 0.23-0.38]. Expenditure on transport was not significantly different between arms. The mean supplementary non-medical out-of-pocket expenditure during the HC visit was US$0.2.

### Treatment cost from the societal perspective

The total cost of treatment of an outpatient with suspected or confirmed malaria was comprised of the cost of diagnosis, drugs dispensed and patient costs. The unit cost of treatment was cheapest in the presumptive arm (Table 5), followed by that in the RDT arm while microscopy was the most expensive.

**Table 5:** Unit cost of outpatient treatment by diagnostic arm (US$)

Cost category	Presumptive	RDT	Microscopy
Unit cost of diagnosis^a^	0.62	1.29	1.53
Mean cost of AL only per visit^b^	1.38	1.38	1.38
Mean cost of antibiotics/analgesics per visit^c^	1.05	1.05	1.05
Patient costs per visit (mean)^d^	1.97	2.37	2.69
Unit cost of OPD treatment visit with AL only^e^	3.97	5.04	5.60
Unit cost of OPD treatment visit with AL+antibiotic/analgesic^f^	5.02	6.09	6.65
Unit cost of OPD treatment visit with antibiotic/analgesic^g^	3.64	4.71	5.27

AL = artemether-lumefantrine, OPD = outpatient department, RDT = rapid diagnostic test, e = (a+b+d), f = (a+b+c+d), g = (a+c+d)

### Determining the incremental cost effectiveness ratio (ICER)

**Figure 1:**
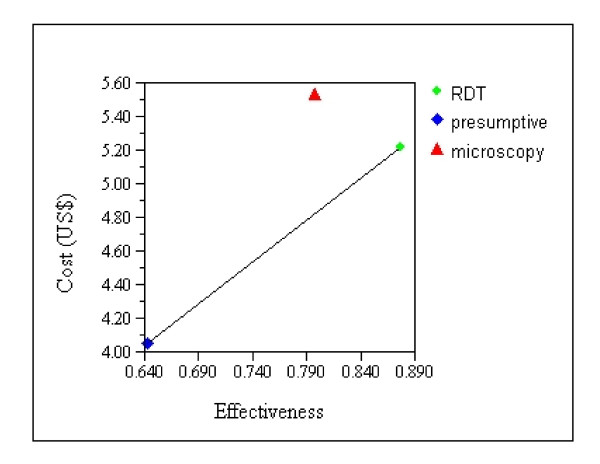
**Cost effectiveness of three malaria diagnostic strategies**.

Using the unit cost per patient correctly diagnosed and treated compared with the effectiveness of each strategy, overall RDT was the most cost effective (Figure 1) with the lowest ICER US$5.0 compared to microscopy US$9.61 (Table 6). In the high transmission setting, the ICER was US$4.38 for RDT and US$12.98 for microscopy. The corresponding ICERs in the low transmission setting were US$5.85 for RDT and US$7.63 for microscopy respectively. The difference in ICERs between RDT and microscopy was greater in the high transmission setting (US$8.9) than in the low transmission area (US$1.78).

**Table 6:** Cost effectiveness of three malaria diagnostic strategies text report

Strategy	Cost (US$)	Incremental Cost (US$)	Effectiveness	Incremental Effectiveness	Average Cost/Effectiveness ratio	ICER
Presumptive	4.04		0.643		6.28	
RDT	5.22	1.17	0.877	0.234	5.95	5.0
Microscopy	5.53	1.48	0.797	0.154	6.94	9.61 (Dominated)
Dominance Report: The strategy "microscopy" is dominated by "RDT".						

ICER = incremental cost-effectiveness ratio, RDT = rapid diagnostic test

### Sensitivity analysis

Overall, a reduction in the cost of AL and RDT was associated with improvement in the cost effectiveness of RDT and microscopy. An increase in malaria prevalence was associated with an improvement in the cost effectiveness of RDT. At a willingness to pay of US$2.8, the RDT remained cost effective up to a threshold value of the cost of treatment of US$4.7.

## Discussion

Following the change in the malaria treatment policy in 2005/2006, the Uganda government commenced the rollout of RDTs in 2008 to target AL to only parasitaemic patients. However, as the health system has struggled with stock-out of RDTs since 2009, the price of AL has decreased from US$2.4 in the year 2004/2005 [2,3,15,16,22,23] to the current average of US$1.38 due to the global campaign to reduce costs and presence of generic products in the market. It was therefore imperative to institute an empirical economic evaluation of the three malaria diagnostic strategies in remote primary care centres in a health system where patients do not pay for medical services.

It is reported here that diagnosing malaria based on signs and symptoms alone (presumptive diagnosis) had the lowest cost (US$0.62). The clinician time was a major recurrent input that determined the cost of presumptive diagnosis. However, the effective clinician contact time was similar in the three arms. Also, the unit cost of outpatient treatment was lowest in the presumptive arm whether the patient got AL alone or in combination with antibiotics. In this analysis, the presumptive method was the base-case because documentation of fever or history of fever has traditionally been considered sufficient evidence for prescribing anti-malarial therapy in rural primary level HCs in Uganda as elsewhere in sub-Saharan Africa. Even when microscopy is available, not all patients get the service, yet clinicians often prescribe anti-malarial treatment to persons with negative microscopy results [7,24,25]. Presumptive diagnosis was the least effective. However, effectiveness of all methods was inversely related to transmission intensity. This observation was also reported in Tanzania [2].

Overall, the RDT technique was the most effective. This finding is supported by comparable studies carried out in other settings [2,15,16], although effectiveness in these studies was determined against microscopy as reference standard. An earlier publication [12] from the current trial reported that expert microscopy "would be gold standard" performed poorly. Here, effectiveness is defined as the proportion of patients correctly diagnosed (true positive + true negative) and treated. Use of expert microscopy as gold standard would erroneously increase the sensitivity and specificity resulting into high effectiveness values. In the current paper therefore, effectiveness was determined with PCR as the reference standard.

The major parameter that determined the cost of RDT-based patient management was cost of the test supplemented by the personnel cost for giving the service. The current price of RDT (US$0.84) at point of use is still high, although it has decreased over time. Previous reports have used RDT costs ranging from US$1.30 to US$1.50 [15,16]. In the current paper, the use of RDT increased the cost of diagnosis by US$ 0.67 (about 108% increase) in comparison with the presumptive technique. An increase in provider cost with introduction of RDTs as a diagnostic service was also reported in Tanzania [2,13]. This increase is however likely to be offset by its superior effectiveness if clinicians adhere to test results. The cost of radical drug treatment normally occurred with all strategies. However, the unit cost of outpatient treatment visit was lower than that of microscopy but higher than in the presumptive method.

Microscopy was less effective compared to RDT. The cost of microscopy diagnostic service was however higher (US$1.53) than that of RDT (US$1.29). In malaria microscopy, the major cost input was the personnel salary constituting 57.5% while laboratory supplies and equipment constituted only 17.9% of the diagnostic service. Laboratory-related capital costs (laboratory space, equipment, etc) were additional costs which were not inputs in the RDT or presumptive arms. This paper focussed on the evaluation of the cost effectiveness of the three strategies at point of care in rural settings. A study in Thailand that extended the analysis to include the pre-service training reported microscopy to be even more expensive [26]. In the Thailand study, however, the presumptive arm was non-existent while blood smears were drawn from patients and transported to static centres for microscopic investigations constraining appropriate comparison with the current findings.

With regard to external costs, the opportunity cost of travel was not significantly different between study arms. The opportunity cost of waiting for treatments especially test results was the major external indirect cost more so in the microscopy arm. The direct cost of transport was not different between study arms, but it was significantly higher in the setting of low transmission due to the hilly difficult terrain. Overall, these external costs were a major part of the unit cost per patient correctly treated and on ICERs.

Few comparable empirical cost-effectiveness analyses done in Africa were available at the time of writing this paper. However, a full comparison with the current findings was difficult. The study in Zambia [15] examined the cost effectiveness of the three strategies from the narrow provider perspective, and was carried out in sentinel sites, which in reality did not represent typical rural HCs. In Tanzania [2], the cost effectiveness of RDT was only compared to microscopy, the presumptive arm was missing. In addition, the study was performed in hospital settings where patient volumes would not be comparable to those attending HCs. On the other hand, in Nigeria [16], patients five years and older paid for the medical services, which impacted on their direct expenditure. Further, the Nigerian study used a different measure of effectiveness and patients were treated with dihydroxy-artemisinin/piperaquine, while costing was based on AL. At the time these studies were conducted, the costs of AL and RDT were higher than the current rates. However other than the study in Tanzania [2], all support the current findings that RDT is the most cost effective.

In all scenarios in the sensitivity analysis, RDT maintained its superior cost effectiveness compared to presumptive diagnosis and microscopy. With the global campaign to reduce the prices of ACT and RDTs, policy makers need to re-think and make contingency plans regarding malaria diagnosis. As the cost of AL decreases, presumptive treatment is likely to become more attractive. This scenario is likely to mirror the era of chloroquine or chloroquine/sulphadoxine-pyrimethamine as first-line drugs that were cheap. Therefore, with falling prices of AL, measures need to be put in place to sensitize the health service users about the benefits of appropriate malaria diagnosis and treatment.

## Conclusion

RDT was the most cost effective. However, with the reduction in the cost of RDT and AL, the Malaria Control Programme and stakeholders need a contingency plan regarding malaria diagnosis. Further, there is need to sensitize health service users about the benefits of appropriate malaria diagnosis.

## Competing interests

The authors declare that they have no competing interests.

## Authors' contributions

All authors conceived and designed the study; VB and FN collected, analysed, interpreted the data and drafted the manuscript; PM and KSH critically revised the manuscript and performed further analysis where necessary. All authors read and approved the final manuscript.
